# Impact of the COVID-19 pandemic in childhood and adolescent cancer care in northern Tanzania: a cross-sectional study

**DOI:** 10.1186/s12885-024-12168-y

**Published:** 2024-04-12

**Authors:** Yotham Gwanika, Hannah E. Rice, Madeline Metcalf, Pamela Espinoza, Happiness D. Kajoka, Henry E. Rice, Catherine Staton, Blandina T. Mmbaga, Esther Majaliwa, Emily R. Smith, Cesia Cotache-Condor

**Affiliations:** 1 Pediatric Hematology and Oncology Services, Kilimanjaro Christian Medical Centre, Moshi, Tanzania; 2 Duke Primary Care, Population Health, Duke University, Durham, NC USA; 3 Duke Global Health Institute, Duke University, Durham, NC USA; 4 Duke Center for Global Surgery and Health Equity, Duke University, Durham, NC USA; 5 Division of Emergency Medicine, Department of Surgery, Duke University Medical Center, Durham, NC USA; 6 Kilimanjaro Clinical Research Institute, Kilimanjaro Christian Medical Centre, Moshi, Tanzania; 7 Kilimanjaro Christian Medical University College, Moshi, Tanzania

**Keywords:** Childhood cancer, Pediatric oncology, Adolescent cancer, COVID-19 pandemic, Northern tanzania, Kilimanjaro

## Abstract

**Introduction:**

The SARS-CoV-2 (COVID-19) pandemic has strained healthcare systems and presented unique challenges for children requiring cancer care, particularly in low- and middle-income countries. This study aimed to assess the impact of the COVID-19 pandemic on access to cancer care for children and adolescents in Northern Tanzania.

**Methods:**

In this cross-sectional study, we assessed the demographic and clinical characteristics of 547 pediatric and adolescent cancer patients (ages 0–19 years old) between 2016 and 2022 using the population-based Kilimanjaro Cancer Registry (KCR). We categorized data into pre-COVID-19 (2016–2019) and COVID-19 (2020–2022) eras, and performed descriptive analyses of diagnostic, treatment, and demographic information. A secondary analysis was conducted on a subset of 167 patients with stage of diagnosis at presentation.

**Results:**

Overall admissions nearly doubled during the pandemic (*n* = 190 versus 357). The variety of diagnoses attended at KCMC increased during the pandemic, with only five groups of diseases reported in 2016 to twelve groups of diseases in 2021. Most patients were diagnosed at a late stage (stage III or IV) across eras, with the proportion of under-five years old patients increasing late-diagnoses from 29.4% (before the pandemic), 52.8% (during the pandemic), when compared to the overall cohort. Around 95% of children in this age category reported late-stage diagnosis during the pandemic. Six out of the twelve cancer site groups also reported an increase in late-stage diagnosis. During the pandemic, the proportion of children receiving surgery increased from 15.8 to 30.8% (*p* < 0.001).

**Conclusion:**

Childhood and adolescent cancer care changed in Northern Tanzania during the COVID-19 pandemic, with increased late-stage diagnoses presentations among younger patients and the increased use of surgical therapies in the context of a growing practice. Understanding the impact of the COVID-19 pandemic on pediatric and adolescent cancer care can help us better adapt healthcare systems and interventions to the emerging needs of children and adolescents with cancer in the midst of a health crisis.

**Supplementary Information:**

The online version contains supplementary material available at 10.1186/s12885-024-12168-y.

## Introduction

Globally, nearly 400,000 children are diagnosed with cancer each year [1, 2]. The prevalence of childhood cancer is largely concentrated in low- and middle-income countries (LMICs), where more than 85% of cancer deaths currently occur [1, 2]. However, oncology services in LMICs remain low on the global health agenda and funding priorities, with just 5% of global cancer spending being allocated to LMICs [3–7]. Since the development of the World Health Organization (WHO) Global Initiative for Childhood Cancer in 2018, more attention has been placed on increasing survival for pediatric cancer patients, with a goal of 60% survival globally by 2030 [8]. 

Health emergencies have historically exposed disparities within the health systems [9]. The emergence of the COVID-19 pandemic highlighted persistent and systemic health inequalities in many areas of children’s health care, especially in LMICs [10, 11]. The morbidity and mortality associated with childhood cancer are directly impacted by the timeliness and consistency of oncology treatment, with many infrastructure, medication access, and other resources disrupted during the COVID-19 pandemic [12–15]. In particular, many LMICs experienced delays in accessing chemotherapy, radiation, and surgical treatments, as well as shortages in blood transfusions and medications needed for cancer care [16]. Systematic disruptions and delays in cancer care for adults during the COVID-19 pandemic have also been noted in several LMICs, with a decrease in patient volume, staff shortages, and modifications of cancer treatment modalities resulting in significant delays and poor health outcomes [14, 17, 18]. 

In Tanzania, 3,000 children are diagnosed with cancer each year, with survival rates estimated between 5 and 10% [2, 19]. Resources and infrastructure for pediatric and adolescent cancer care were limited in Tanzania prior to the pandemic, with only one pediatric oncologist serving the entire northern region of Tanzania [20, 21]. Pumphrey et al. reported disruptions in adult cancer care with increased mortality rates in Tanzania, along with increased visits to traditional healers and extended delays in treatment [22]. Our understanding of pediatric and adolescent oncology service delivery and outcomes in Tanzania during the pandemic remains poorly understood. In this study using an established cancer registry, we compared children’s cancer diagnoses, treatments, and outcomes before and during the COVID-19 pandemic with the aim of assessing the impact of the COVID-19 pandemic in the access of cancer care at one of the only medical centers serving pediatric and adolescent patients within the Kilimanjaro region of Tanzania.

## Methods

### Study setting

In 1971, the Kilimanjaro Christian Medical Centre (KCMC) was established as one of four zonal referral hospitals in Tanzania. KCMC serves patients from the northern and surrounding regions of Tanzania, providing care for approximately 11 million patients each year [23]. KCMC is one of just two cancer centers providing oncology care for both adult and pediatric patients in northern Tanzania. KCMC currently hosts one pediatric surgeon and one of the six pediatric oncologists in the country. Oncology services offered at KCMC include diagnosis, treatment, research, and community programming. KCMC utilizes 12 chemotherapy bays offering standard chemotherapy services, 47 inpatient beds, 4 consultation rooms, and an onsite laboratory. Through the financial support of KCMC and philanthropic organizations, families incur no direct medical costs for pediatric oncology treatments and services at KCMC.

### Data collection

We collected data for this study from the Kilimanjaro Cancer Registry (KCR). The KCR, a member of the African Cancer Registry Network (AFCRN), was developed in 1998 and is the longest standing population-based cancer registry in Tanzania. In 2016, the KCR began the inclusion of child and adolescent cancer cases in the registry. The KCR collects data from oncology patients across the five districts within the Kilimanjaro region (Moshi Urban, Moshi Rural, Rombo, Hai, and Sija). Registry data are thoroughly reviewed by the cancer registrar and assistant registrar monthly to ensure validity of data.

Our data included children and adolescents from the KCR aged 0–19 years with newly diagnosed cancer between January 2016 and December 2022. We collected demographic variables, including age, sex, travel time to KCMC, and geographic region of referral categorized as either the ‘Kilimanjaro Region’ or ‘Other Region.’ Travel time was estimated in hours using the driving option from Google Maps. Clinical variables included diagnosis, treatment, stage of cancer upon diagnosis, and vital status (alive, dead, unknown) at the last point of clinical contact.

Cancer diagnoses were divided into twelve site groups according to the International Classification of Childhood Cancer, third edition (ICCC-3): Group I, Leukemias, myeloproliferative diseases, myelodysplastic diseases; Group II, Lymphomas and reticuloendothelial neoplasms; Group III, CNS and miscellaneous intracranial and intraspinal neoplasms; Group IV, Neuroblastoma and other peripheral nervous cell tumors; Group V, Retinoblastoma; Group VI, Renal tumors; Group VII, Hepatic tumors; Group VIII, Malignant bone tumors, Group IX, Soft tissue and other extraosseous sarcomas; Group X, Germ cell tumors, trophoblastic tumors, and neoplasms of gonads; Group XI, Other malignant epithelial neoplasms and malignant melanomas; and Group XII, Other and unspecified malignant neoplasms [24]. 

Stage of cancer at the time of diagnosis (I, II, III, IV) was available for 167 of the 547 participants. Although comparison of staging for different childhood cancer types is complicated by the large number of staging systems used for individual cancer types (i.e. TNM system for solid tumors, SIOP system for Wilms Tumor, hematological staging systems, etc.), we categorized all cancer types as Stage I-IV by following the staging conversion recommended for cancer registries in LMICs as detailed in the AFCRN *Childhood Cancer Staging Rules for Population Based Registries* manual [25]. Long-term follow-up data were not available due to the recent addition of pediatric patients into the KCR.

### Statistical analyses

We stratified all registry subjects into two time periods, a pre-COVID-19 era (2016–2019) and a COVID-19 era (2020–2022). Descriptive statistics were used to compare the demographic and clinical characteristics between the Pre-COVID-19 and during the COVID-19 era, which were further summarized by age group. We used the World Health Organization classification for age [26]. Inferential statistical analysis was used to compare all variables by year of diagnosis and disease stage at diagnosis for both periods. Receiving surgery before and during the COVID-19 pandemic was further compared across all cancer diagnosis groups excluding hematological malignancies. Categorical variables were assessed at the 95% significance level (*p* < 0.05) using chi-square statistical analysis. A secondary analysis was conducted on a sample of 167 children for whom there was available data on the stage of disease at the time of diagnosis. All data analyses were conducted with Stata v15.1 (StataCorp, College Station, TX).

## Results

Between 2016 and 2022, 547 cancer patients between 0 and 19 years old were registered in the KCR and were included in this study. Overall, the frequency of new admissions nearly doubled from before the COVID-19 pandemic (*n* = 190) to during the COVID-19 pandemic (*n* = 357) (Table 1). The distribution of age differed between before the pandemic and during the pandemic (*p* = 0.022). During the pandemic, there was a higher proportion of patients aged 5–9 years (18.9% *n* = 36/190 before the pandemic, 25.8% *n* = 92/357 during the pandemic) and patients aged 10–14 (17.9% *n* = 34/190 before the pandemic, 21.0% 75/357 during the pandemic). In contrast, the proportion of patients aged 15–19 years old decreased during the pandemic (before the pandemic 27.9% *n* = 53/190, 17.4% during the pandemic *n* = 62/357). The proportion of patients under five years old was similar before and during the pandemic.

**Table 1:** Demographic and clinical characteristics before and during the COVID-19 Pandemic (*n* = 547)

Variable	Total	Pre-COVID-19 Era (2016–2019)	COVID-19 Era (2020–2022)	P-value
	n (%) 547 (100.0)	n (%) 190 (34.8)	n (%) 357 (65.2)	
**Age (years)**				
00–04	195 (35.6)	67 (35.3)	128 (35.9)	
05–09	128 (23.4)	36 (18.9)	92 (25.8)	**0.022**
10–14	109 (19.9)	34 (17.9)	75 (21.0)	
15–19	115 (21.0)	53 (27.9)	62 (17.4)	
**Sex**				
Male	319 (58.3)	109 (57.4)	210 (58.3)	
Female	228 (41.7)	81 (42.6)	147 (41.7)	0.742
**Region**				
Kilimanjaro	208 (38.0)	78 (40.8)	130 (36.4)	
Other	339 (62.0)	112 (58.6)	227 (63.6)	0.287
**Time travel**				
Less than 1 h	141 (25.8)	55 (29.0)	86 (24.0)	
Between 2–5 h	338 (61.8)	114 (60.0)	224 (62.8)	0.421
More than 6 h	68 (12.4)	21 (11.0)	47 (13.2)	
**Treatment**				
*Radiotherapy*				
No	507 (92.7)	177 (93.2)	330 (92.4)	
Yes	40 (7.3)	13 (6.8)	27 (7.6)	0.758
*Chemotherapy*				
No	103 (18.8)	28 (14.7)	75 (21.0)	
Yes	444 (81.2)	162 (85.3)	282 (79.0)	0.074
*Surgery*				
No	407 (74.4)	160 (84.2)	247 (69.2)	
Yes	140 (25.6)	30 (15.8)	110 (30.8)	**< 0.001**
**Diagnosis**				
Group I	126 (23.0)	38 (20.0)	88 (26.4)	
Group II	122 (22.3)	42 (22.1)	80 (22.4)	
Group III	26 (4.8)	8 (4.2)	18 (5.0)	
Group IV	8 (1.5)	3 (1.6)	5 (1.4)	
Group V	80 (14.6)	24 (12.6)	56 (15.7)	0.793
Group VI	72 (13.2)	30 (15.8)	42 (11.8)	
Group VII	7 (1.3)	2 (1.1)	5 (1.4)	
Group VIII	30 (5.5)	13 (6.8)	17 (4.8)	
Group IX	45 (8.2)	17 (8.9)	28 (7.8)	
Group X	8 (1.5)	4 (2.1)	4 (1.1)	
Group XI	15 (2.7)	7 (3.7)	8 (2.2)	
Group XII	1.5 (8)	2 (1.1)	6 (1.7)	
**Date of last contact**				
Alive	436 (79.7)	150 (81.1)	286 (80.1)	
Dead	106 (19.4)	35 (18.9)	71 (19.9)	**0.008**
Unknown	5	5	0	

*Notes* Group I: Leukemias, myeloproliferative diseases, myelodysplastic diseases; Group II: Lymphomas and reticuloendothelial neoplasms; Group III: CNS and miscellaneous intracranial and intraspinal neoplasms; Group IV: Neuroblastoma and other peripheral nervous cell tumors; Group V: Retinoblastoma; Group VI: Renal tumors; Group VII: Hepatic tumors; Group VIII: Malignant bone tumors, Group IX: Soft tissue and other extraosseous sarcomas; Group X: Germ cell tumors, trophoblastic tumors, and neoplasms of gonads; Group XI: Other malignant epithelial neoplasms and malignant melanomas; Group XII: Other and unspecified malignant neoplasms

Group I diseases (leukemias, myeloproliferative diseases, myelodysplastic diseases) and group II diseases (lymphomas and reticuloendothelial neoplasms) were the most common diagnoses in both eras (Table 1). In the pre-COVID-19 era, 20.0% (*n* = 38/190) of patients were diagnosed with group I diseases whereas 22.1% (*n* = 42/190) were diagnosed with group II diseases. In the COVID-19 era, 26.4% (*n* = 88/357) of the cases were in the group I category while 22.4% (*n* = 80/357) were in the group II category. Group V (retinoblastoma) and group VI (renal tumors) were the next most frequent diagnoses in both periods.

**Fig. 1:**
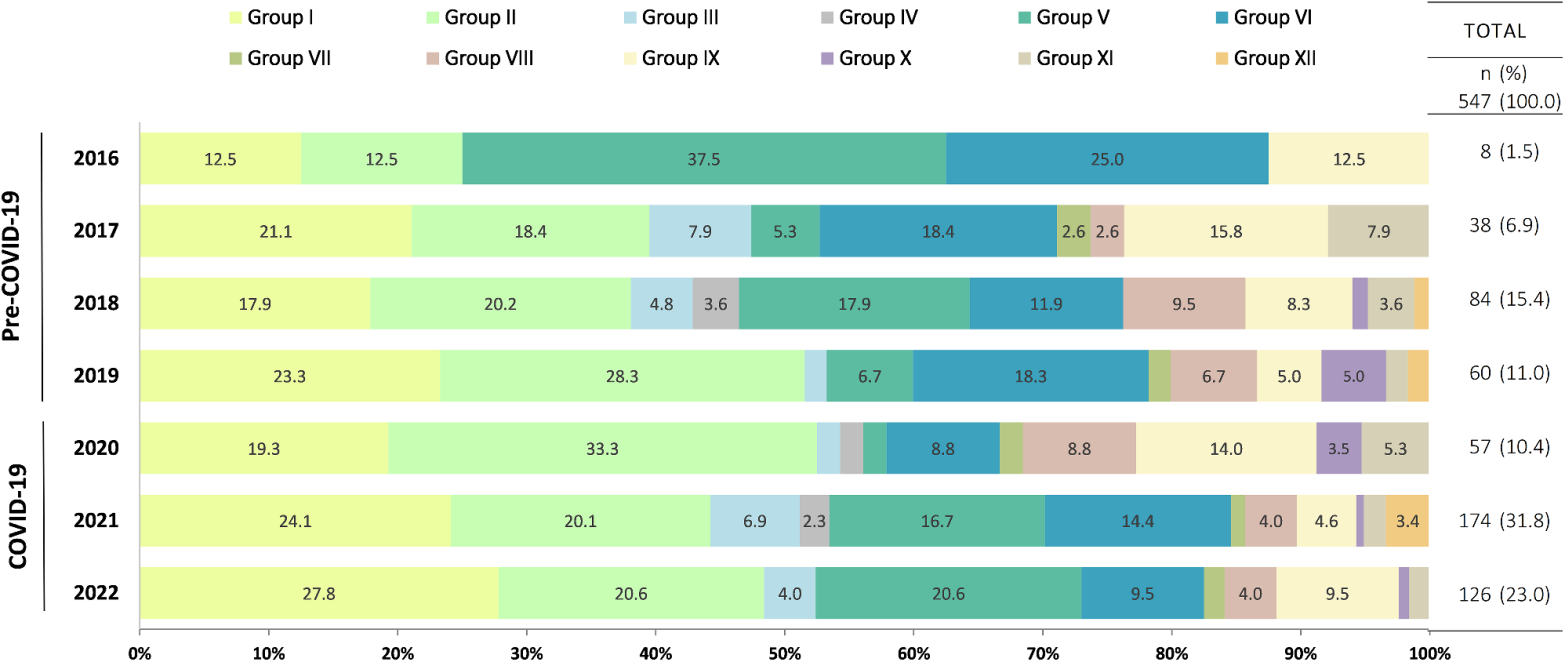
Distribution of diagnosis by site groups and across all years, from 2016 to 2022 (*n* = 547). *Notes* Site groups with no available percentage data in the figure reported less than 2% of total cases for the respective year. The exact value was not included because of space limitations. Group I: Leukemias, myeloproliferative diseases, myelodysplastic diseases; Group II: Lymphomas and reticuloendothelial neoplasms; Group III: CNS and miscellaneous intracranial and intraspinal neoplasms; Group IV: Neuroblastoma and other peripheral nervous cell tumors; Group V: Retinoblastoma; Group VI: Renal tumors; Group VII: Hepatic tumors; Group VIII: Malignant bone tumors, Group IX: Soft tissue and other extraosseous sarcomas; Group X: Germ cell tumors, trophoblastic tumors, and neoplasms of gonads; Group XI: Other malignant epithelial neoplasms and malignant melanomas; Group XII: Other and unspecified malignant neoplasms

The frequency of new admissions increased from 2016 (*n* = 8) to 2022 (*n* = 126), with a brief decrease between 2019 (*n* = 60) and 2020 (*n* = 57) (Fig. 1). The stratification of diagnoses by year also showed an increase in the variety of diagnoses attended at KCMC from before the pandemic to during the pandemic, with only five groups of diseases reported in 2016 to twelve groups of diseases in 2021. The proportion of patients diagnosed with cancer groups I, V, VI, and X decreased from before the pandemic to during the pandemic when compared to the overall cohort, but the frequencies within each group increased. For instance, in 2016 (before the pandemic), 37.5% (*n* = 3/8) of the cases were diagnosed with retinoblastoma. This proportion fell to 1.8% (*n* = 1/57) in 2020 during the COVID-19 pandemic. However, the proportion of retinoblastoma cases increased to 16.7% (*n* = 29/174) in 2021 and 20.6% (*n* = 26/126) in 2022. The proportion of patients diagnosed with cancer groups II (lymphomas and reticuloendothelial neoplasms), VIII (malignant bone tumors), and XI (soft tissue and other extraosseous sarcomas) increased from before the pandemic to during the pandemic. Although pediatric and adolescent oncology admissions to KCMC decreased from its peak of 84 patients in 2018 to 57 patients in 2020 at the beginning of the pandemic, an increase of patients was observed in 2021 (*n* = 174) and 2022 (*n* = 126).

**Fig. 2:**
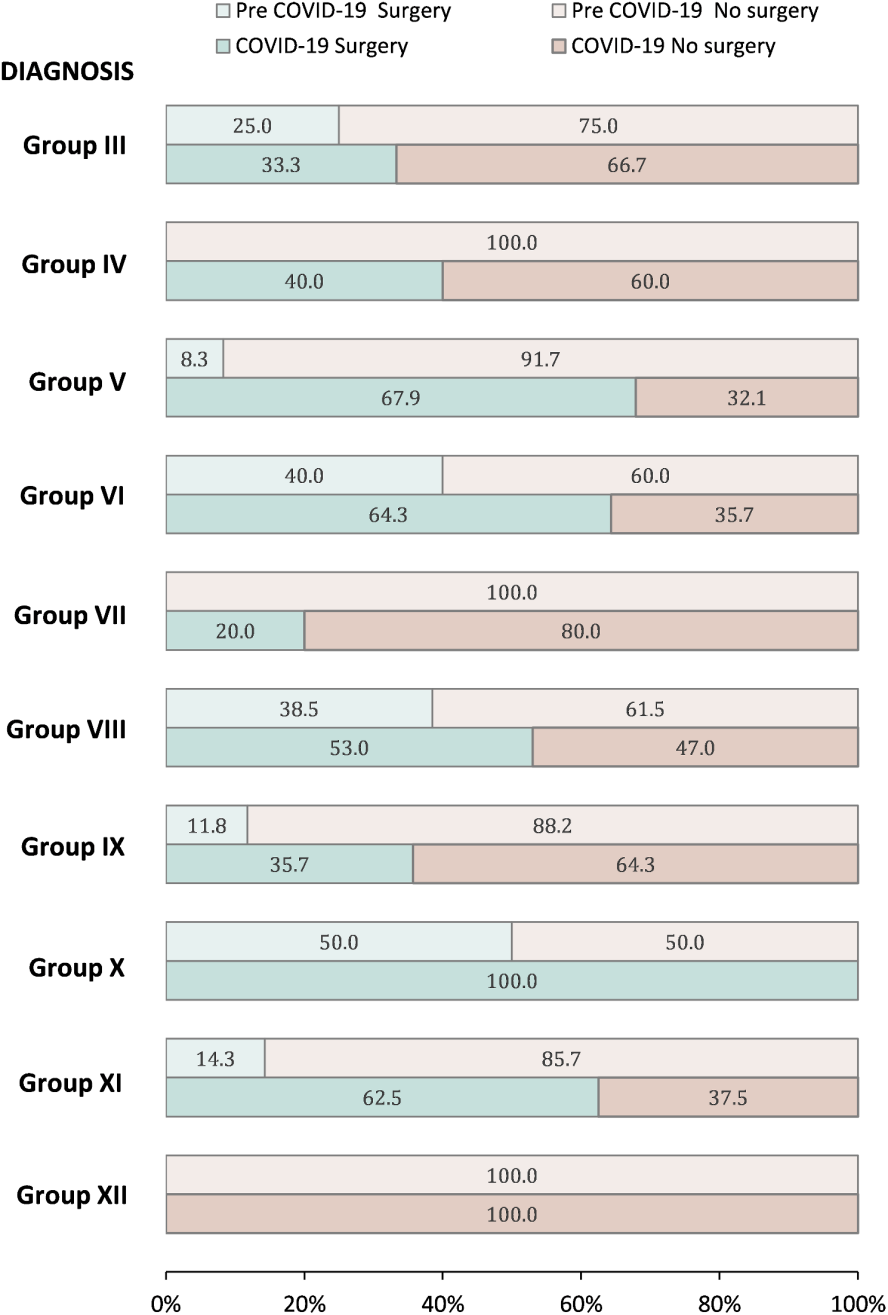
Distribution of surgery (yes/no) stratified by cancer site group (III-XII) before and during COVID-19. *Notes* Group III: CNS and miscellaneous intracranial and intraspinal neoplasms; Group IV: Neuroblastoma and other peripheral nervous cell tumors; Group V: Retinoblastoma; Group VI: Renal tumors; Group VII: Hepatic tumors; Group VIII: Malignant bone tumors, Group IX: Soft tissue and other extraosseous sarcomas; Group X: Germ cell tumors, trophoblastic tumors, and neoplasms of gonads; Group XI: Other malignant epithelial neoplasms and malignant melanomas; Group XII: Other and unspecified malignant neoplasms

The types of treatment modalities changed during the COVID-19 pandemic. The proportion of children undergoing chemotherapy was similar across eras, with 85.3% *n* = 162/190 (before the pandemic) and 79.0% *n* = 282/357 (during the pandemic) (*p* = 0.074) (Table 1). The proportion of children undergoing radiotherapy was also similar between eras with 6.8% *n* = 13/190 (before the pandemic) and 7.6% *n* = 27/357 (during the pandemic) (*p* = 0.758). The proportion of children undergoing surgery increased from 15.8% *n* = 30/190 (before the pandemic) to 30.8% *n* = 110/357 (during the pandemic) (*p* < 0.001), with a constant increase across all years (Additional file 1). When surgery (yes/no) was stratified by diagnosis (except groups I and II) and compared before and during the COVID-19 pandemic, we found all diagnosis groups experienced an increase in the proportion of patients undergoing surgical care. Groups V (from 8.35 to 67.9%), group X (from 50.0 to 100.0%), and group XI (from 14.3 to 62.5%) showed the greatest increase from before the pandemic to during the pandemic. Only diagnosis group XII did not show any increase with no patients undergoing surgery before and during the COVID-19 pandemic (Fig. 2 and Additional file 2).

The stage of cancer upon diagnosis was recorded for a subset of patients (*n* = 167) (Table 2). Although most patients presented at a late stage (stage III or IV) before (77.3%, *n* = 68/88) and during (84.8%, *n* = 67/79) the COVID-19 pandemic, the patterns of stage at presentation changed during the pandemic. The number of children less than five years of age with stages III & IV increased 1119 from 29.4% (*n* = 20/68) before the pandemic to 52.8% (*n* = 39/67) during the pandemic, while the proportion of patients of all other ages decreased during the pandemic. Around 95% (*n* = 39/41) of children less than five years old were diagnosed at stages III & IV during the pandemic and the proportion of late-stage diagnoses ranged from 60 to 80% for the other age categories despite the mentioned decrease compared to before the pandemic (*p* = 0.029).

**Table 2:** Stage of diagnosis before and during the COVID-19 Pandemic (*n* = 167)

Variable	Pre-COVID- 19 Era (2016–2019)		P-value	COVID-19 Era (2020–2022)		P-value
	Stage I, II n (%) 20 (22.7)	Stage III, IV n (%) 68 (77.3)		Stage I, II n (%) 12 (15.2)	Stage III, IV n (%) 67 (84.8)	
**Age**						
00–04	5 (25.0)	20 (29.4)		2 (16.7)	39 (58.2)	
05–09	6 (30.0)	13 (19.1)	0.764	3 (25.0)	12 (17.9)	**0.029**
10–14	3 (15.0)	10 (14.7)		4 (33.3)	6 (9.0)	
15–19	6 (30.0)	25 (36.8)		3 (25.0)	10 (14.9)	
**Sex**						
Male	12 (60.0)	31 (45.6)	0.257	7 (58.3)	39 (58.2)	0.994
Female	8 (40.0)	37 (54.4)		5 (41.7)	28 (41.8)	
**Region**						
Kilimanjaro	8 (40.0)	33 (48.5)	0.501	6 (50.0)	21 (31.3)	0.210
Other	12 (60.0)	35 (51.5)		6 (50.0)	46 (68.7)	
**Time travel**						
Less than 1 h	5 (25.0)	21 (30.9)		5 (41.7)	11 (16.4)	
Between 2–5 h	13 (65.0)	39 (57.4)	0.828	5 (41.7)	47 (70.1)	0.104
More than 6 h	2 (10.0)	8 (11.8)		2 (16.7)	9 (13.4)	
**Treatment**						
*Radiotherapy*						
No	17 (85.0)	64 (94.1)	0.185	12 (100.0)	57 (85.1)	0.152
Yes	3 (15.0)	4 (5.9)		0 (0.0)	10 (14.9)	
*Chemotherapy*						
No	2 (10.0)	7 (10.3)	0.970	1 (8.3)	12 (17.9)	0.410
Yes	18 (90.0)	61 (89.7)		11 (97.1)	55 (82.1)	
*Surgery*						
No	17 (85.0)	53 (77.9)	0.491	7 (58.3)	35 (52.2)	0.697
Yes	3 (15.0)	15 (22.1)		5 (41.7)	32 (47.8)	
**Date of last contact**						
Alive	17 (85.0)	47 (69.1)		11 (91.7)	52 (77.6)	
Dead	3 (15.0)	19 (27.9)	0.340	1 (8.3)	15 (22.4)	0.265
Unknown	0	2		0	0	

**Fig. 3:**
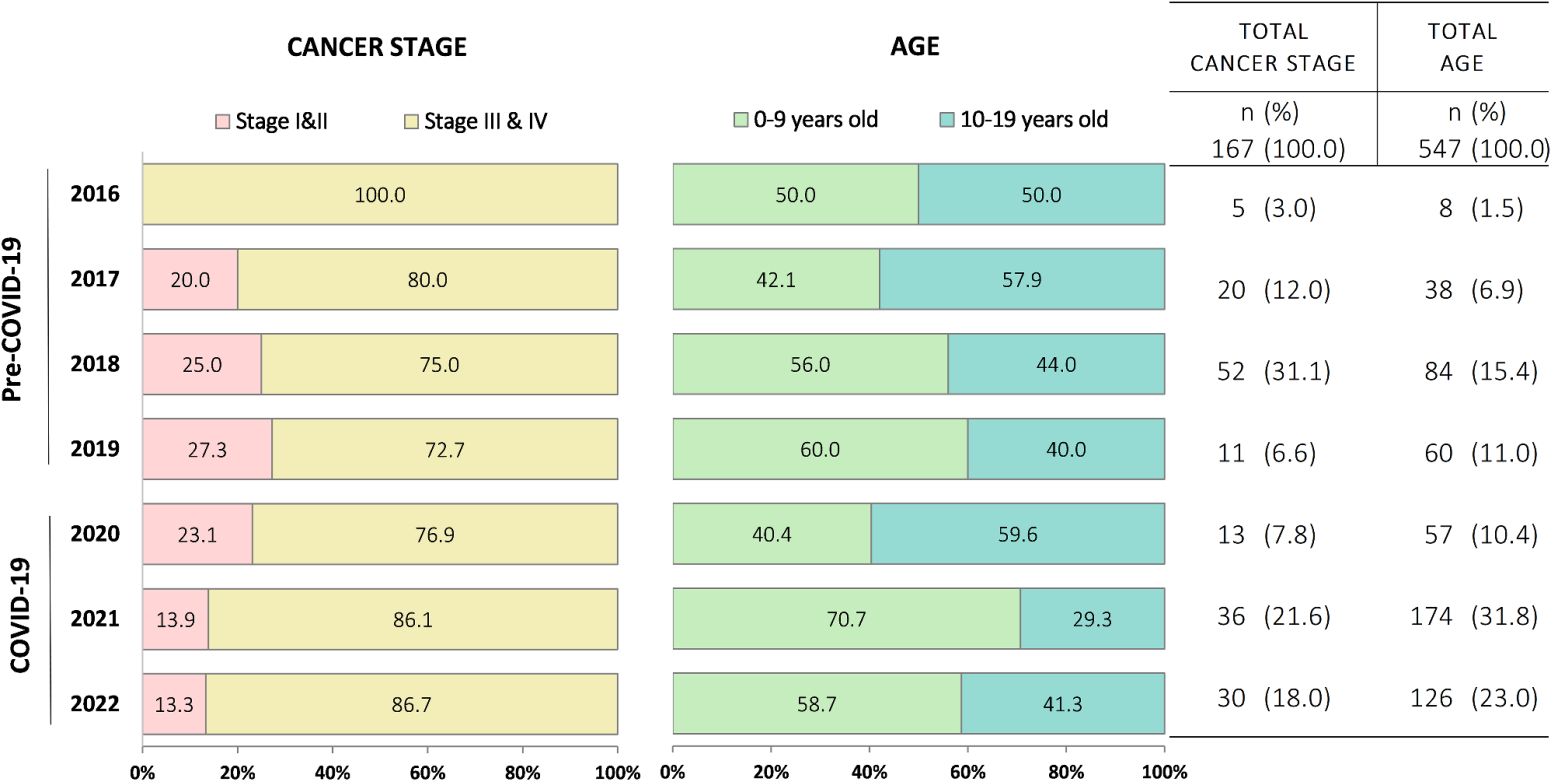
Distribution of cancer cases by stage (*n* = 167) and age group (*n* = 547) before and during COVID-19

**Fig. 4:**
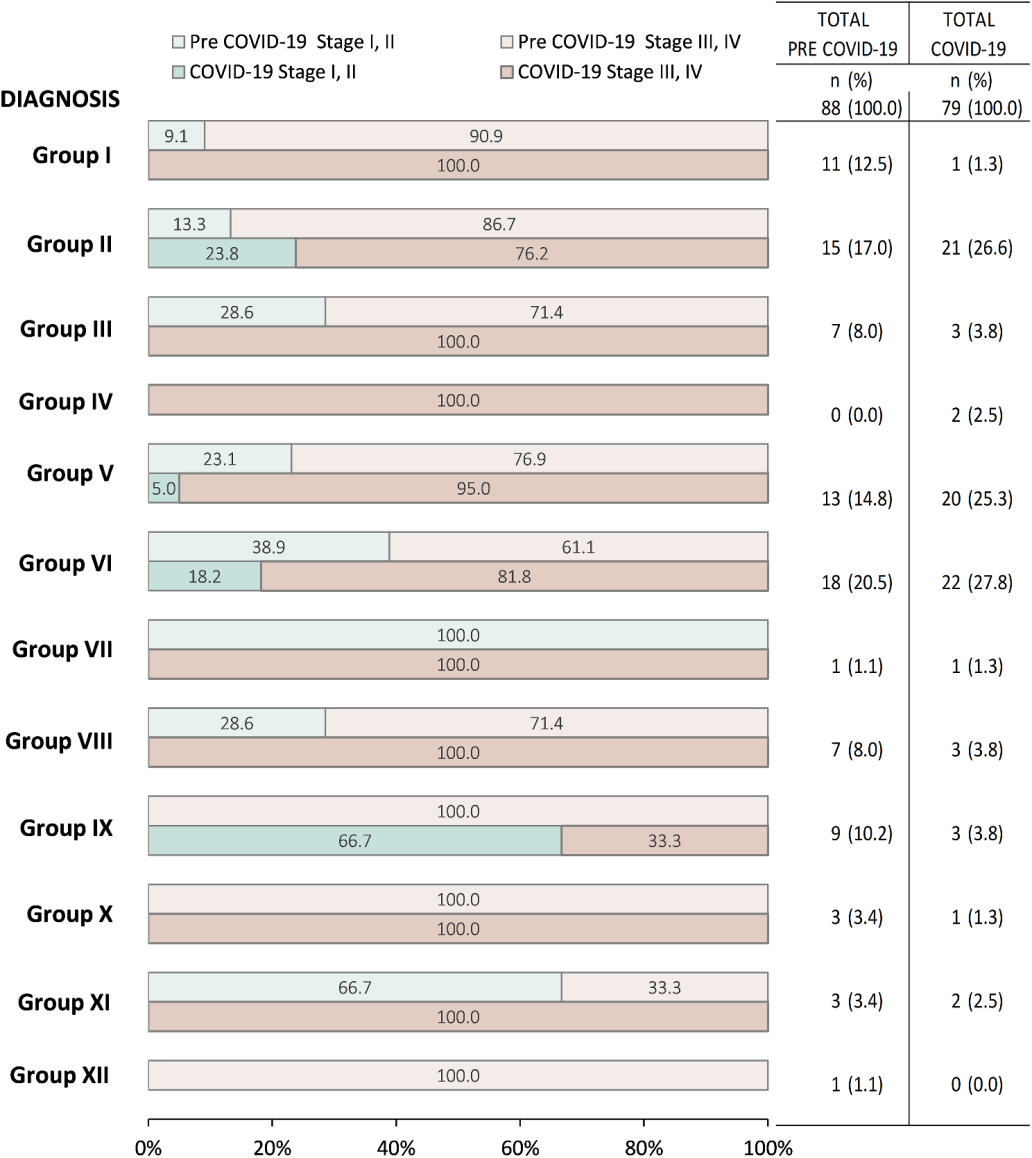
Distribution of diagnosis by site groups and across stage before and during COVID-19 (*n* = 167). *Notes* Group I: Leukemias, myeloproliferative diseases, myelodysplastic diseases; Group II: Lymphomas and reticuloendothelial neoplasms; Group III: CNS and miscellaneous intracranial and intraspinal neoplasms; Group IV: Neuroblastoma and other peripheral nervous cell tumors; Group V: Retinoblastoma; Group VI: Renal tumors; Group VII: Hepatic tumors; Group VIII: Malignant bone tumors, Group IX: Soft tissue and other extraosseous sarcomas; Group X: Germ cell tumors, trophoblastic tumors, and neoplasms of gonads; Group XI: Other malignant epithelial neoplasms and malignant melanomas; Group XII: Other and unspecified malignant neoplasms

A further stratification of age and stage by year suggested that 2021 was the year with the highest peak of increase in admissions of patients aged 0–9 and decrease of admissions in patients aged 10–19 (Fig. 3). During the pre-COVID era, there was a decrease in the proportion of patients diagnosed at stages III & IV, from 100% (*n* = 5/5) in 2016 to 72.7% (*n* = 8/11) in 2019. With the onset of the pandemic, this trend reversed reaching up 86.1% (*n* = 31/36) in 2022. During the pandemic, the proportion of patients diagnosed at stage III & IV increased in groups I, III, V, VI, VIII, and XI increased compared to prior years (Fig. 4). Leukemias, myeloproliferative diseases, myelodysplastic diseases increased from 90.9% (*n* = 10/11) to 100.0% (*n* = 1/1). CNS and miscellaneous intracranial and intraspinal neoplasms increased from 71.4% (*n* = 5/7) to 100.0% (*n* = 3/3). Retinoblastomas increased from 76.9% (*n* = 10/13) to 95.0% (*n* = 19/20). Renal tumors increased from 61.1% (*n* = 11/18) to 81.8% (*n* = 18/22). Malignant bone tumors increased from 71.4% (*n* = 5/7) to 100.0% (*n* = 3/3). Other malignant epithelial neoplasms and malignant melanomas increased from 33.3% (*n* = 1/3) to 100.0% (*n* = 2/2).

## Discussion

In this cross-sectional and registry-based study, we found some changes in the diagnosis and treatment patterns of childhood and adolescent cancer patients at KCMC during the COVID-19 pandemic. Overall, new admissions nearly doubled compared to pre-pandemic numbers. With the onset of the pandemic, under-five years old patients saw an increase in their already high proportion of late-stage presentations, giving this age category the highest burden of cases diagnosed at a late stage. The proportion of admissions in four cancer site groups decreased during the pandemic and late diagnosis increased in six out of the twelve cancer site groups. Treatment patterns also varied during the pandemic with an increase in surgical procedures. Our findings constitute a foundational step both to understand how cancer care was impacted during the pandemic and how to design specific strategies to manage such changes in future health emergencies.

Our research revealed a significant rise in total admissions throughout the pandemic period. Despite changes in diagnosis patterns, including a decrease in the proportion of certain cancer types compared to the overall cohort, new admissions increased across the majority of diagnosis groups during this time. This phenomenon distinguishes KCMC, as other institutions reported a decline in pediatric cancer patient admissions amid the COVID-19 pandemic [27]. Several factors might explain this phenomenon. First, Tanzania’s minimal lockdown policy during the pandemic might have initially caused delays in seeking care for children but families eventually arrived at KCMC. Second, the increasing reputation of KCMC as a specialized children’s cancer center and the presence of the region’s only pediatric oncologist might have led to an increase in referrals and more families choosing KCMC for treatment despite the challenges posed by the pandemic [21]. Finally, KCMC’s elimination of direct out-of-pocket expenses and the provision of comprehensive support for pediatric cancer patients (i.e., food, transportation, and education) since 2021 might have played a positive role in the decision-making process of families to seek care for their children [21]. Our findings suggest the need to reimagine policies and interventions tailored to expanding healthcare facilities, so cancer systems in these places can successfully build resilience and go through the preparation, response, and recovery phases during a global health crisis.

Our findings are also consistent with previous studies that have shown that the COVID-19 pandemic has disproportionally affected and aggravated existing disparities among pediatric patients with cancer in LMICs [28]. The pandemic has posed multiple barriers to cancer care, resulting in long delays in diagnosis or treatment of pediatric patients [16, 27, 29–31]. The increase in the proportion of under-five-years-old patients being diagnosed at a late stage while the remaining age groups maintained over 60% of late-stage diagnoses speaks to a persistent and, in some cases, aggravated issue of delays in accessing cancer care in northern Tanzania. Travel bans and quarantine measures restricted patients from reaching timely care in Tanzania [27, 30, 32]. In addition to preexisting barriers to accessing cancer care, including financial distress, travel times, lack of information, and weak referral systems; [33] different reasons have been suggested to explain longer delays in the decision of caregivers to seek care for their children, including an increased priority for COVID-19 patients (KCMC was designated as the regional COVID-19 treatment center), restrictions in public transportation, border lockdowns, as well as the anxiety of contracting the virus during their stay at the medical facilities [16, 17, 27, 30, 32]. Also, regulations in African settings during the pandemic required the suspension of early cancer diagnosis programs, impacting directly the number of available surveillance consultations [16, 34]. Early diagnosis is crucial for childhood cancer patients [15, 32]. The swift initiation of treatment can prevent the development of metastases, improve quality of life, and increase the chances of survival for children; all factors which were impacted with the onset of the COVID-19 pandemic [12, 13, 29, 35]. 

Consistent with findings in other LMICs, children at KCMC presented with later-stage cancers during the COVID-19 pandemic. Other studies reported similar patterns of disease presentation, with an increase in the proportion of patients diagnosed with advanced stages of the disease during the pandemic [29, 36, 37]. In our study, this migration from early-stage to late-stage cancer diagnosis was observed in half of the twelve ICCC-3 cancer groups (I, III, V, VI, VIII, and XI). Although these results reveal interesting findings, they should be taken with caution as the sample size for the cohort with stage available data is limited.

Previous studies conducted among African health systems reported disruptions of different cancer care services [34]. However, our study shows that childhood and adolescent cancer care was available and supply chains were not substantially disrupted at KCMC. Although surgical rates for pediatric cancer decreased in many LMICs during the pandemic [27], surgical procedures for most of the diagnosis groups where surgical care is indispensable increased at KCMC during the pandemic. This may in part be due to many areas of elective surgical services being paused during the pandemic, and paradoxically surgical teams had the capacity to conduct more emergency surgical procedures, including cancer-related surgical treatments. This increase in procedures is also related to the overall increase in admissions despite the challenges posed by the pandemic. This phenomenon underscores a paradoxical trend that might be evident in expanding healthcare facilities such as KCMC, where there is a heightened demand for additional resources dedicated to cancer treatment and surgical interventions amidst a global infectious disease emergency.

As one of the few medical centers providing pediatric and adolescent cancer care in Tanzania, KCMC has demonstrated consistent progress in expanding the quality and accessibility of cancer care services for children and adolescents within the Kilimanjaro region. Prior to the COVID-19 pandemic, KCMC had made notable strides in decreasing the prevalence of late-stage diagnoses of pediatric and adolescent cancer. However, the several disparities in the access of timely cancer care worsened with the onset of the pandemic, particularly for children under-five and 15–19 years old patients. We advocate for an urgent change in policy that favors community- and family-oriented strategies to equip families with the knowledge and resources needed to navigate their children’s cancer care even amid public health emergencies. Failure to address these issues may have long-lasting consequences for pediatric and adolescent cancer patients in the region, particularly during health system stressors such as future pandemics.

### Limitations

Although we provide a comprehensive overview of the impact of the COVID-19 pandemic on pediatric oncology care and outcomes, our study contains some limitations. First, although our data were obtained from a validated pediatric cancer registry, we are faced with the limited scope of a retrospective cross-sectional study design, including lack of causal inference, and limited generalizability of study results. Second, we acknowledge the limitation of staging data and the lack of appropriate staging data for a significant amount of the study sample. Data availability remains a challenge in LMICs and low-resource settings due to the overall disruptions in cancer treatment and diagnosis during the COVID-19 pandemic, therefore the collection of this data may have been further impacted [17, 22, 38]. Additional research is needed to assess the effect of the pandemic specifically on pediatric and adolescent cancer data availability, reporting, and registry operations. Finally, absconding rates, lost-to-follow-up, and progression of disease due to lack or discontinuity of treatment are some factors that could have contributed to the assessment of the impact of the COVID-19 pandemic on cancer care at KCMC. However, such data was not included in the registry.

## Conclusion

Our study has observed an increase in late-stage cancer diagnosis in young children, decrease in admissions of adolescent patients and changes in treatment modalities in northern Tanzania during the COVID-19 pandemic. Understanding how the COVID-19 pandemic impacted existing challenges and disparities in the access to cancer care for children and adolescents can help us to build more resilient healthcare systems that efficiently respond to the needs of children and adolescents suffering from cancer in the midst of a health crisis.

### Electronic supplementary material

Below is the link to the electronic supplementary material.

Supplementary Material 1

Supplementary Material 2

## Data Availability

The datasets generated and/or analyzed during the current study are not publicly available because they are curated by the Kilimanjaro Cancer Registry but are available from the corresponding author on reasonable request.

## Abbreviations

LMICs: middle-income countries
WHO: World Health Organization
KCMC: Kilimanjaro Christian Medical Centre
KCR: Kilimanjaro Cancer Registry
ACRN: African Cancer Registry Network
ICCC-3: International Classification of Childhood Cancer, third edition
