# Supplementary material for: Impact of the COVID-19 pandemic in childhood and adolescent cancer care in northern Tanzania: a cross-sectional study
Source: BMC Cancer. 2024 Apr 12;24:457. doi: 10.1186/s12885-024-12168-y (PMC11010397; doi:10.1186/s12885-024-12168-y)
Supplement: Supplementary file 1 — Supplementary Material 1 [file 12885_2024_12168_MOESM1_ESM.docx]

**Additional file 2.** **Distribution of surgery across diagnosis groups III-XII before and after the COVID-19 pandemic.**

| **Before COVID-19 pandemic (2016-2019)** | | | | | | | | |
| --- | --- | --- | --- | --- | --- | --- | --- | --- |
| **Cancer groups (excluding hematological malignancies)** | **Total**  *% (n)*  *100.0 (110)* | | **No**  *% (n)*  *76.4 (84)* | | **Yes**  *% (n)*  *23.6 (26)* | | **P-value** | |
| CNS and miscellaneous intracranial and intraspinal neoplasms | 8 (4.2) | | 6 (3.8) | | 2 (6.7) | | 0.105 | |
| Neuroblastoma and other peripheral nervous cell tumors | 3 (1.6) | | 3 (1.9) | | 0 (0.0) | |  |  |
| Retinoblastoma | 24 (12.6) | | 22 (13.8) | | 2 (6.7) | |  |  |
| Renal tumors | 30 (15.8) | | 18 (11.3) | | 12 (40.0) | |  |  |
| Hepatic tumors | 2 (1.1) | | 2 (5.0) | | 0 (0.0) | |  |  |
| Malignant bone tumors | 13 (6.8) | | 8 (5.0) | | 5 (16.7) | |  |  |
| Soft tissue and other extraosseous sarcomas | 17 (8.9) | | 15 (9.4) | | 2 (6.7) | |  |  |
| Germ cell tumors, trophoblastic tumors, and neoplasms of gonads | 4 (2.1) | | 2 (1.3) | | 2 (6.7) | |  |  |
| Other malignant epithelial neoplasms and malignant melanomas | 7 (3.7) | | 6 (3.8) | | 1 (3.3) | |  |  |
| Other and unspecified malignant neoplasms | 2 (1.1) | | 2 (1.3) | | 0 (0.0) | |  |  |
| **During COVID-19 pandemic (2020-2022)** | | | | | | | | |
| **Cancer groups (excluding hematological malignancies)** | | **Total**  *% (n)*  *100.0 (189)* | | **No**  *% (n)*  *46.0 (87)* | | **Yes**  *% (n)*  *54.0 (102)* | | **P-value** |
| CNS and miscellaneous intracranial and intraspinal neoplasms | | 18 (5.0) | | 12 (4.9) | | 6 (5.5) | | 0.002 |
| Neuroblastoma and other peripheral nervous cell tumors | | 5 (1.4) | | 3 (1.2) | | 2 (1.8) | |  |
| Retinoblastoma | | 56 (15.7) | | 18 (7.3) | | 38 (34.5) | |  |
| Renal tumors | | 42 (11.8) | | 15 (6.1) | | 27 (24.5) | |  |
| Hepatic tumors | | 5 (1.4) | | 4 (1.6) | | 1 (0.9) | |  |
| Malignant bone tumors | | 17 (4.8) | | 8 (3.2) | | 9 (8.2) | |  |
| Soft tissue and other extraosseous sarcomas | | 28 (7.8) | | 18 (7.3) | | 10 (9.1) | |  |
| Germ cell tumors, trophoblastic tumors, and neoplasms of gonads | | 4 (1.1) | | 0 (0.0) | | 4 (3.6) | |  |
| Other malignant epithelial neoplasms and malignant melanomas | | 8 (2.2) | | 3 (1.2) | | 5 (4.5) | |  |
| Other and unspecified malignant neoplasms | | 6 (1.7) | | 6 (2.4) | | 0 (0.0) | |  |
