# Supplementary figures and images for: Impact of the COVID-19 pandemic in childhood and adolescent cancer care in northern Tanzania: a cross-sectional study
Source: BMC Cancer. 2024 Apr 12;24:457. doi: 10.1186/s12885-024-12168-y (PMC11010397; doi:10.1186/s12885-024-12168-y)

**Additional file 1.** **Distribution of cancer treatment by year, from 2016 to 2022 (n=547).**


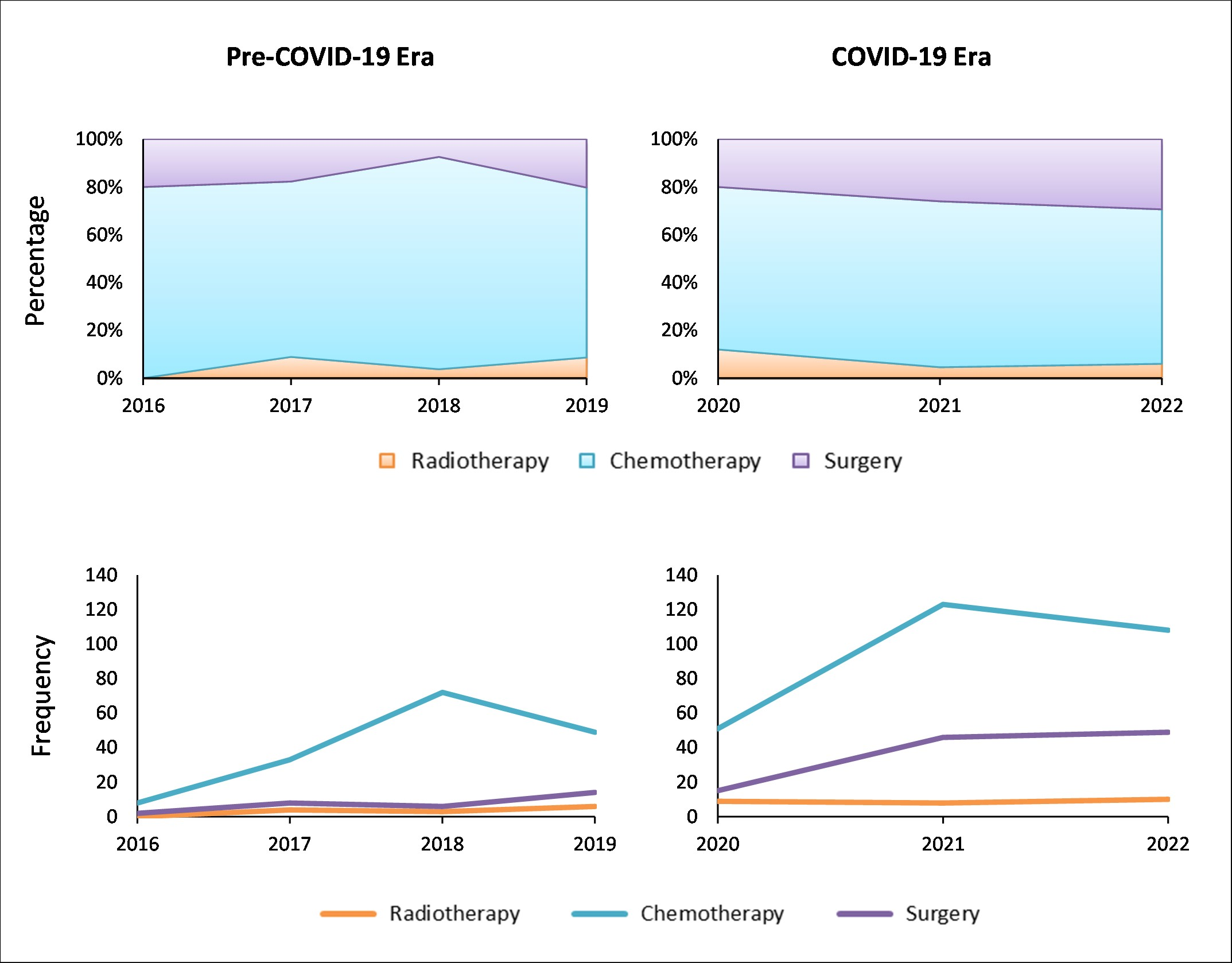

Supplement: Supplementary file 2 — Supplementary Material 2 [file 12885_2024_12168_MOESM2_ESM.docx]
